# Osteomesopyknosis: A Bone-Sclerosing Disorder that May be Confused with Bone Metastases

**DOI:** 10.1007/s00223-025-01361-1

**Published:** 2025-03-14

**Authors:** José A. Riancho, Alvaro del Real, Ana I. Vega, Rosa Landeras, Anibal Ferrerira, Ana C. Ferreira, Jose Sainz, Remedios Quirce, Eva Martínez de Castro

**Affiliations:** 1 Servicio de Medicina Interna, Hospital U.M. Valdecilla, Universidad de Cantabria, Av Valdecilla Sn, 39008 Santander, Spain; 2 Universidad de Cantabria, Santander, Spain; 3 Instituto de Investigación Marqués de Valdecilla (IDIVAL), Santander, Spain; 4 Centro de Investigación Biomédica en Red de Enfermedades Raras (CIBERER), Madrid, Spain; 5 Servicio de Genética, Hospital U.M. Valdecilla, Santander, Spain; 6 Servicio de Radiodiagnóstico, Hospital U.M. Valdecilla, Santander, Spain; 7 Servicio de Nefrología, Hospital Curry Cabral, Lisbon, Portugal; 8 LADICIM, Universidad de Cantabria, Santander, Spain; 9 Servicio de Medicina Nuclear, Hospital U.M. Valdecilla, Santander, Spain; 10 Servicio de Oncología, Hospital U.M. Valdecilla, Santander, Spain

**Keywords:** Bone sclerosis, Bone metastases, Cancer, Osteomesopyknosis

## Abstract

Sclerosing bone disorders encompass a range of genetic and acquired diseases. The potential for bone metastases is often a significant concern, especially when multiple discrete lesions are present. Several non-malignant disorders can also produce similar patterns of bone abnormalities. Among these is osteomesopyknosis, a rare condition characterized by multiple sclerotic lesions in the axial skeleton. Only a few cases of this presumably genetic disease have been documented. In this report, we present a new case and review previously published cases to enhance understanding and facilitate recognition of this disorder within the broader category of sclerosing bone diseases.

## Introduction

Many tumors cause skeletal lesions, osteolytic, osteoblastic, or mixed. Several non-malignant disorders can also cause similar lesions. The diagnosis may be difficult, particularly in the case of multifocal osteosclerotic lesions, which may be confused with metastases and hinder proper decision-making (1,2). Among the hereditary bone-sclerosing diseases, osteopetrosis is well-known [1–3]. Others, such as osteomesopyknosis, may be less well-recognized and be a cause of diagnostic confusion, particularly with bone metastases [4]. Here we report a new case of osteomesopyknosis and review previously published cases to achieve a more profound comprehension of the disease.

### Case Report

A 65-year-old woman was sent to our clinic because of sclerosing bone lesions. Eleven months earlier she began to feel epigastric pain and diagnostic studies performed at another hospital revealed a poorly differentiated gastric adenocarcinoma. Radiographic studies revealed multiple sclerosing skeletal lesions that were attributed to metastases. Hence, radical therapy was dismissed and systemic chemotherapy and denosumab (four doses of 120 mg over 10 months) were indicated. A few months later she was sent to our hospital for re-evaluation.

**Fig. 1:**
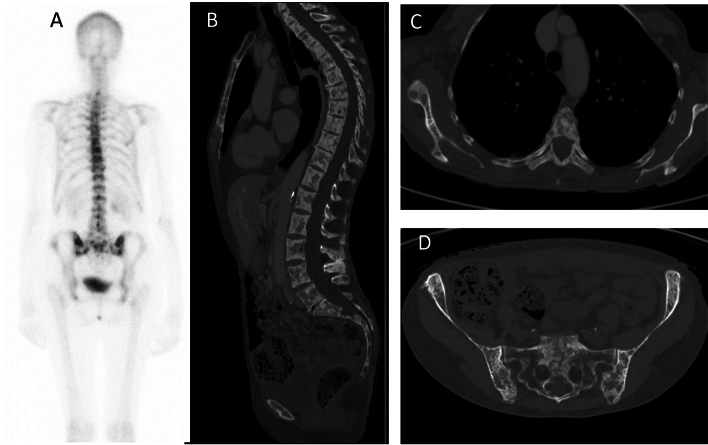
Imaging studies. **A** 99mTc-DPD scintigraphy showed multiple foci of increased uptake: **B–D** CT scan

She had no musculoskeletal symptoms. Her past medical history, family history, and physical exam were unremarkable. A DPD-Tc99m bone scan showed multiple foci of increased uptake at the spine, ribs, sacrum, and iliac bones (Fig. 1A). A pelvic CT scan confirmed multiple, predominantly blastic, lesions affecting the pelvic bones and proximal femora. An iliac biopsy revealed non-specific changes and the absence of neoplastic cells.

**Fig. 2:**
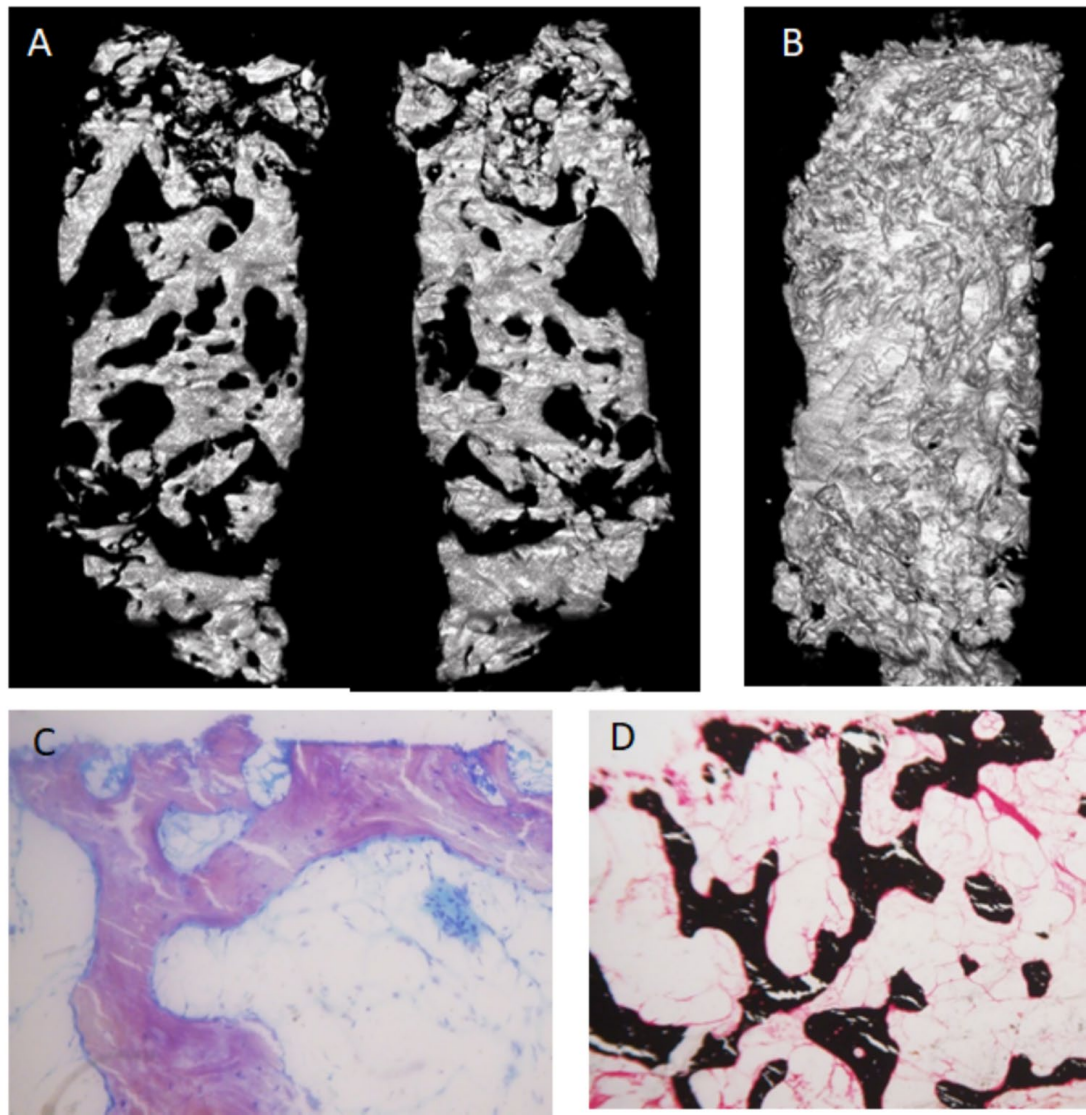
Bone biopsy. **A** Micro-Ct scan, two representative sagittal reconstructions; **B**Whole volume reconstruction. Histological analysis (**C**, Toluidine blue staining × 16; D, Von Kossa × 6) shows low turnover (with some inactive lining cells, no osteoclasts) and normal mineralization. It is to note that the biopsy was obtained after several denosumab infusions

Fasting blood chemistries, including, creatinine, calcium, phosphate, alkaline phosphatase, albumin, protein electrophoresis, tryptase, and 25-hydroxyvitamin D were all normal. PTH was slightly elevated (140 pg/ml, normal < 88). Bone turnover markers were low (PINP 20 ng/ml, CTX 0.04 ng/ml). These results were considered secondary to previous denosumab therapy. DXA showed z-scores of + 1.2 at the lumbar spine, −0.7 at the femoral neck, −0.9 at the total hip, −2.4 at ultra-distal radius, and −1.9 at the mid-radius. Another CT obtained four months later did not reveal significant changes (Fig. 1B–D). CT-guided bone biopsy was repeated, and two new cylinders of the iliac bone were obtained. One was decalcified and subjected to conventional histological analysis. Again, there were no malignant cells. Micro-CT of the other sample showed a marked increase in trabecular bone, with trabecular bone volume/total volume of 30.3% (Fig. 2A–B). This is well above the 18–21% values reported in several studies of normal subjects [5, 6]. The fragment then underwent undecalcified histology. The study was limited by the poor quality of the sample and previous denosumab therapy. Nevertheless, it showed lines of inactive osteoblasts – lining cells – in some portions of the trabecular bone, without current osteoid formation. The osteoid surface appeared increased, albeit impossible to quantify, without an increase in its thickness. No osteoclasts were observed (Fig. 2C–D). Currently, it appeared as a low remodeling bone disease.

Given the radiographic images, histology, and the absence of progression, we established the diagnosis of osteomesopyknosis. A whole exome sequencing analysis did not reveal pathogenic/likely pathogenic variants in skeleton-related genes. Due to the absence of metastases, an elective gastric resection was performed. Unfortunately, the patient experienced postoperative complications not related to bone metabolism and died soon after surgery.

## Discussion

Sclerosing bone disorders include a variety of genetic and acquired diseases. Bone metastases are particularly important among the latter. Other diseases associated with more or less generalized bone sclerosis include fluoride intoxication, systemic mastocytosis, renal osteodystrophy. They are usually excluded by medical and epidemiological history and biochemical studies [1, 2]

The diagnostic approach of patients with sclerosing bone disorders requires an integrative approach combining epidemiological data, family history, clinical manifestations, and ancillary data, particularly biochemical and imaging studies. In this regard, it is useful to establish if osteosclerosis affects diffusely the skeleton or is limited to one or a few bones. Likewise, it is important to determine if it affects spongy bone (osteosclerosis), cortical bone (hyperostosis), or both.

The osteopetroses are the most typical disorders with generalized osteosclerosis, affecting both axial and appendicular bones with obliteration of marrow spaces [7]. Different from those with osteopetrosis, some patients present sclerotic lesions predominantly in the axial skeleton, such as the spine and the pelvis. Some frequent causes of axial sclerosis are listed in Table 1.

**Table 1:** Selected causes of axial osteosclerosis

- Hereditary
o Without skeletal dysplasia
▪ Normal alkaline phosphatase
• Osteomesopyknosis
▪ Increased alkaline phosphatase
• Axial osteomalacia
• Hyperostosis autosomal dominant with high Alkaline Phosphatase
o With skeletal dysplasia (usually with cranial or long bone lesions)
• Osteopetrosis
• Pyknodysostosis
• Van Buchen disease and sclerosteosis
• Other (some cases of osteopathia striata, osteopoikilosis, fibrogenesis imperfecta, etc.)
- Acquired
o Infections
o Metabolic and toxic: renal osteodystrophy, fluorosis
o Inflammatory and other conditions: Chronic recurrent multifocal osteomyelitis, histiocytosis, mastocytosis, myelofibrosis, sickle cell disease, sarcoidosis, Paget’s disease, bone infarcts, bone islands
o Tumors: metastases, myeloma/POEMS, lymphoma, primary bone tumors (osteoid osteoma, osteoblastoma, osteosarcoma)

When osteosclerotic lesions are detected in adult patients, the possibility of malignancy is a major concern, especially if, as in the case reported here, the patient has a tumor that can metatasize to bone. Tumors of the prostate and the breast most commonly cause osteoblastic metastases. Other carcinomas (including those of the gastrointestinal tract, pancreas, and urinary system), lymphomas, carcinoid tumors, neuroblastomas, and medulloblastomas may occasionally produce osteoblastic lesions [2]. Although radiological features may often help to establishing the correct diagnosis, the work-up may be complicated, particularly in cases of multifocal lesions. Bone biopsy may be needed to exclude metastases. A CT-guided procedure is usually preferred. However, the reliability for excluding cancer is usually lower in sclerotic lesions (reported diagnostic yield 67–74%) than in lytic lesions (diagnostic yield 88%) [8–10].

Osteomesopyknosis (MIM 166450, ORPHA 2777) is a rare form of axial osteosclerosis. Only a few cases have been reported (Table 2). It is a benign disorder, and most patients show only minor symptoms, such as mild back pain. Since it is a common non-specific symptom, it may be difficult to attribute it to the disease with certainty. In some patients, sclerotic lesions may be an incidental finding in imaging studies. Typically, it appears as multiple sclerotic lesions within the trabecular regions of the axial skeleton. Lesions can be seen in plain radiographs, but are better defined by CT or MRI. As in the case reported here, the lesions are metabolically active and show high uptake of several radionuclides used in isotopic scans. Serum calcium, phosphate, alkaline phosphatase, and other chemistries are usually normal. About 20 cases of different families have been reported in the literature. All cases typically show sclerosing lesions of the spine and the pelvis. Patients may also have lesions at the proximal femora and humeri (either radiological or in bone scans). Rare cases show lesions in more distal bones or scoliosis. Most patients have multifocal discrete osteosclerotic lesions. The well-defined margins and symmetrical distribution of the lesions, as well as the absence of periostal reaction or associated lytic lesions, may help establish their non-neoplastic origin [2, 11, 12]. However, some show more diffuse osteosclerosis. A “rugger jersey”-like pattern may be present in the vertebrae. The mechanical integrity of bone appears conserved, and fractures have not been reported.

**Table 2:** Osteomesopyknosis cases

Author, yr	Index patients	Country	Age	Ethnic/Race	Sex	Known family cases	Manifestations	Location	Biopsy	Other
Simon, 1979 [22]	1	France	26		Female	Yes	Pain	Spine, pelvis		
Maroteaux 1980 [17]	5	France	30		Male		Pain	Spine, pelvis, proximal femur		Femoral geode
		France	10		Male		Scoliosis	Spine, pelvis		
		France	16		Female		Pain	Spine, pelvis		Mild rethrognatia
		France	15		Male			Spine, pelvis		Slightly decreased height of dorsal vertebrae
		France	17		Male			Spine, pelvis		Slightly decreased height of dorsal vertebrae, Small iliac wings
Stoll 1981 [18]	1	France	27		Female	Mother, brother	None	Spine, pelvis		
Proschek 1985 [23]	1	Canada	14		Male	Father, brother	Pain	Spine, pelvis, proximal femur		
Griffith 1988 [24]	1	UK	14	European	Male	No*	Pain	Spine, pelvis, proximal femur		Rugger jersey, diffuse sclerosis
Delcambre 1989 [25]	2	France	16	European	Female	Mother, 2 brothers	Pain	Spine, pelvis, proximal femur		Diffuse sclerosis
		France	16	European	male	Mother	Scoliosis	Spine, pelvis, proximal femur		Diffuse sclerosis
Renowden 1992 [26]	1	UK	56	European		Mother, brother, nephews (*)		Spine,pelvis, proximal fémur, humerus, clavicle, scapulae		
Hardouin 1994 [27]	1	France	60	European	Male	Father, brother, daughter	Pain	Spine, pelvis, proximal fémur and humerus	Trabecular thickening, low bone turnover	
Heursen 2016 [4]	1	Spain	34		Male	Sister	Pain	Spine, pelvis	trabecular thickening (sister)	
De la Hoz, 2008 [28]	1	Spain	30		Female	Brother, father	Pain	Spine, pelvis proximal and mid femur, proximal humerus	Trabecular thickening	
Madruga 2012 [20]	1	Portugal	32	European	Female		Pain?	Spine, pelvis	Trabecular thickening	
Yao 2014 [29]	1	USA	NR	European	Female	Son	Pain	Spine, pelvis		
Jeoung, 2015 (14)	1	Korea	16	Asian	Female		Pain	Spine		Rugger jersey
Fernández-Luna 2024 [21]	1	Spain	49	European	Female		Pain	Spine, pelvis, proximal femur and humerus	Trabecular thickening, consistent with low turnover	
Present report	1	Spain	65	European	Female		None	Spine, pelvis, ribs	Trabecular thickening, consistent with low turnover	Stomach cancer
Cases with a questionable diagnosis										
Schmidt 1989 [13]	1	Germany	NR		Female	Yes (several) with deformities	Pain	Spine, pelvis proximal femur	Osteomalacia	
Kozlowski 1994 [14]	2	Australia	13		Female					Neuromuscular disorder, dysmorphism
		Bulgaria	13		Male					Dysmorphic features
Quintana, 2005 [16]	1	Colombia	33	Latin American	Female		Pain	Spine, pelvis, scapula, humerus, proximal and distal femur		High AlkPhos, litiasis, hypercalciuria,
Sundaram 2022 [15]	1	USA	31		Male		Tracheal and arterial compression due to clavicular enlargement	NR		Neuromuscular disorder, avascular necrosis of the hip

Extra skeletal manifestations are absent in the vast majority of osteomesopyknosis cases. Among the reported cases of osteomesopyknosis (see Table 2), some have atypical features, including severe bone deformities in the family [13], undiagnosed neuromuscular disorder, and dysmorphic features [14, 15]. Thus, we feel that these patients may indeed have a different disease that includes osteosclerosis among its manifestations (see Table 2). Although osteomesopyknosis and axial osteomalacia have some common features, the latter typically is accompanied by high serum alkaline phosphatase and signs of osteomalacia in bone biopsies. Thus, cases reported by Quintana [16] and Schmidt [13] do not fit within the typical profile of osteomesopyknosis either. Among the remaining 20 cases, both sexes were equally distributed. Patients’ age varied between 10 and 65 years. Thus, bone abnormalities seem to start during the growth period. However, there are no follow-up studies, and the lifetime evolution is unknown.

Given the lack of evidence of acquired causes, a hereditary origin of osteomesopyknosis has been suspected. In many cases, family studies were not done or were incomplete. Nevertheless, several reports of familial cases across multiple generations strongly suggest an autosomal dominant transmission [17, 18]. Ten of the fifteen index cases reported originated from France, Spain, or Portugal. Whether this geographical accumulation of cases truly reflects a higher prevalence in that area is currently unknown.

The mechanisms leading to bone sclerosis are also unknown. However, the normal levels of bone turnover markers and the paucity of osteoblasts and osteoclasts in the few cases with bone biopsies [19–21], suggest that the increased bone tissue is the consequence of decreased bone resorption, rather than increased osteoblastic bone formation. This is also consistent with the biopsy findings in our patient. Unfortunately, the absence of prior tetracycline labeling and the poor sample quality, along with ongoing denosumab therapy, precluded gaining further insight into the baseline remodeling status of the patient from the biopsy and bone turnover markers.

Despite the familiar aggregation of osteomesopyknosis, the involved genes have not been systematically studied. To our knowledge, there is only a single published report with genetic data [21]. That case, identified by our group, was associated with a variant in the *ALOX5* gene that appeared to decrease the RANKL/OPG ratio and consequently reduce osteoclast generation and bone resorption. However, in the present case, exome sequencing did not reveal relevant variants in *ALOX5* or other bone-related genes, thus suggesting genetic heterogeneity.

In summary, we report a new case of osteomesopyknosis, with extensive ancillary studies, including exome analysis, and review the previous reports to better delineate the clinical spectrum of the disease. The case report highlights that although osteomesopyknosis is a benign disorder, it may cause significant confusion, particularly in patients with cancer. “All that glitters is not gold,” and all osteoblastic lesions are not metastases. Hence, clinicians should be aware of this condition to adopt the appropriate diagnostic and therapeutic decisions.
